# Deoxythymidylate Kinase (DTYMK)-Driven Pyrimidine Hypermetabolism Is Associated with a Folate-Dependent Macrophage Programme in Triple-Negative Breast Cancer

**DOI:** 10.3390/ijms27198626

**Published:** 2026-09-26

**Authors:** Gaoge Peng, Dequan Liu, Wucheng Zhu, Beibei Xin, Yi Wu, Yifeng Hou, Hefen Sun, Wei Jin

**Affiliations:** 1 Key Laboratory of Breast Cancer in Shanghai, Fudan University Shanghai Cancer Center, Shanghai 200032, China; 2 Department of Oncology, Shanghai Medical College, Fudan University, Shanghai 200032, China

**Keywords:** triple-negative breast cancer, DTYMK, FOLR2^+^ macrophages, metabolic crosstalk, multi-omics

## Abstract

Immune checkpoint blockade benefits only a minority of patients with triple-negative breast cancer (TNBC), and the tumour-intrinsic programmes that establish its immunosuppressive microenvironment remain poorly defined. Pyrimidine metabolism supplies nucleotides and one-carbon units in malignant cells, but whether it shapes the immune microenvironment is unknown. Integrating single-cell, bulk breast cancer transcriptomics with Xenium spatial transcriptomics, DTYMK knockdown, tumour-conditioned-medium co-culture and folate rescue, we found that pyrimidine pathway activity was elevated in the malignant epithelium of TNBC, within which we identified Epi4, a high-potency subpopulation coupling pyrimidine and folate metabolism and predicted to export folate. Furthermore, this folate was predicted to be taken up by FOLR2^+^ tumour-associated macrophages (TAMs) and was associated with S-adenosylmethionine(SAM)-dependent methylation signatures and MAFB activity. Spatially, Epi4 and FOLR2^+^ TAMs co-localised at the tumour–stroma margin, a region of reduced T-cell density. DTYMK depletion reduced tumour fitness and attenuated folate-mediated macrophage reprogramming, which was largely restored by exogenous folate. Here, we therefore identify a DTYMK–folate–MAFB axis in which tumour metabolic rewiring is associated with epigenetic reprogramming of macrophages.

## 1. Introduction

Breast cancer is the most prevalent malignancy among women globally [1]. Although emerging immunotherapies have transformed oncology, their clinical efficacy in breast cancer remains severely limited [2]. This intrinsic resistance is primarily orchestrated by a highly immunosuppressive tumor microenvironment (TME) that effectively abrogates effector T cell infiltration and cytotoxicity [3].

Metabolic reprogramming physically and biochemically sculpts this immunosuppressive niche through nutrient competition, extracellular acidification, severe hypoxia, and the local accumulation of toxic metabolites [4,5]. Tumor-intrinsic metabolic rewiring acts as the central driver of this niche remodeling; beyond fueling unrestrained cancer cell proliferation, it exerts profound paracrine effects, hijacking the transcriptional programs of adjacent stromal and immune cells to foster a pro-tumorigenic habitat [6,7]. Despite the growing appreciation of metabolic crosstalk between cancer cells and neighboring stromal/immune compartments, the precise cellular subsets orchestrating these metabolic interactions and the underlying mechanisms driving collective tumor progression remain largely elusive [8]. Consequently, decoding these critical cell-to-cell metabolic communications holds immense translational potential for uncovering novel therapeutic vulnerabilities and dismantling the symbiotic armor of the tumor. Historically, metabolic oncology has predominantly centered on glycolysis (lactate production), lipid metabolism, or amino acid dependencies [4,8]. However, the immunomodulatory role of pyrimidine metabolism—an indispensable hub catering to the insatiable demand for DNA synthesis and epigenetic regulation in malignant cells—within the TME has been strikingly overlooked, leaving a critical gap in our comprehensive understanding of metabolically driven immune evasion. Deoxythymidylate kinase (DTYMK) catalyses the phosphorylation of dTMP to dTDP and lies at the convergence of the de novo and salvage pyrimidine biosynthetic routes [9,10]; whether it shapes the breast cancer immune microenvironment has not been established.

Here, by integrating single-cell and high-resolution spatial transcriptomics to decode the immunosuppressive TME in breast cancer, we identified Epi4, a highly malignant tumor epithelial subpopulation defined by hyperactive pyrimidine-folate metabolic flux and profound stemness. We reveal that Epi4 leverages this fundamental metabolic rewiring not solely as an internal biomass reservoir, but as a potent intercellular communication axis. Through the folate network, Epi4 metabolically polarizes adjacent macrophages into an immunosuppressive FOLR2^+^ state, directly sculpting a spatially restricted “immune barrier” niche that physically segregates effector T cells. Collectively, our findings elucidate how a metabolically defined tumor subpopulation constructs an immunosuppressive niche via direct metabolic crosstalk, uncovering a mechanistic vulnerability to dismantle the tumor-supportive microenvironment.

## 2. Results

### 2.1. Subsection

#### 2.1.1. Single-Cell Landscape Reveals a Dominant Epithelial-to-Myeloid Communication Axis in the Tumor Microenvironment

To systematically dissect cellular heterogeneity in breast cancer (BRCA), we analyzed single-cell RNA sequencing (scRNA-seq) data derived from the GSE176078 cohort, mapping the transcriptomic landscape across diverse molecular subtypes (Figure 1A,E).

First, we identified highly expressed genes within each cluster using FindAllMarkers and robustly validated the identities of nine major cellular compartments through canonical marker genes (Figure 1B–D). To elucidate the multicellular ecosystem driving tumor progression, we employed CellChat to infer cell–cell communication networks. Interaction network analysis unveiled extensive global crosstalk between Cancer Epithelial cells and the stromal compartment, particularly involving Myeloid cells, cancer-associated fibroblasts (CAFs), and Endothelial cells (Figure 1F). Subsequent 2D quantification of global interaction strength (incoming vs. outgoing signaling) highlighted a distinct topological hierarchy: Cancer Epithelial cells functioned as the dominant signaling hubs (“Senders”) orchestrating the microenvironment, whereas Myeloid cells and other immune populations served as prominent signal receivers (Figure 1G). Furthermore, pathway-level signaling pattern analysis corroborated this highly directional crosstalk (Figure 1H). This pronounced epithelial-to-myeloid communication axis necessitated a deeper investigation into the specific metabolic and molecular mechanisms driving this critical intercellular dialogue.

#### 2.1.2. Metabolic Profiling Uncovers Pyrimidine Hypermetabolism as a Defining Epithelial Hallmark in TNBC

To decode the overarching metabolic vulnerabilities driving the BRCA microenvironment, we quantified the functional state of core metabolic pathways across all identified cellular compartments using the scMetabolism framework. Global metabolic profiling revealed profound heterogeneity within the tumor ecosystem; notably, the cancer epithelial compartment was distinctly characterized by hyperactive nucleotide synthesis, with pyrimidine metabolism exhibiting the most striking enrichment (Figure 2A).

Spatial projection via UMAP and single-cell quantitative scoring further corroborated that this pyrimidine hypermetabolism is predominantly observed in malignant epithelial cells (Figure 2B,C).

To rigorously evaluate the magnitude of this metabolic rewiring, we calculated Cohen’s d effect sizes, which identified oxidative phosphorylation, alongside pyrimidine and purine metabolism, as the top upregulated pathways in cancer epithelia compared to their normal counterparts (Figure 2D). When these malignant cells were grouped by BRCA molecular subtype, pyrimidine pathway scores were highest in triple-negative breast cancer (TNBC) samples (Figure 2E). Finally, bulk transcriptomic validation utilizing the The Cancer Genome Atlas (TCGA) Breast Invasive Carcinoma (BRCA) cohort cohort independently confirmed the significant global upregulation of pyrimidine metabolism in tumor tissues versus normal adjacent samples (Figure 2F). Collectively, these analyses indicate that elevated pyrimidine pathway activity is a feature of the malignant epithelium in this cohort.

#### 2.1.3. Identification of Epi4, a High-Potency, Hyper-Proliferative Subpopulation Driven by Coupled Pyrimidine and Folate Dependencies

To dissect the functional heterogeneity within the cancer epithelial compartment, we performed unsupervised sub-clustering, revealing five distinct transcriptional states (Epi1–Epi5) (Figure 3A–C and Figure S1A).

Among these, the Epi4 subcluster exhibited a distinct and highly aggressive biological profile. Functional landscape mapping via Gene Ontology (GO) enrichment and Gene Set Variation Analysis (GSVA) unveiled that Epi4 is uniquely driven by the dual activation of proliferation and metabolic machineries (Figure 3D and Figure S1B). Specifically, this subpopulation displayed robust enrichment for cell cycle-driving signatures—including “E2F Targets,” “G2M Checkpoint,” and “MYC Targets”—alongside a profound metabolic reprogramming skewed towards “Pyrimidine Metabolism” and the “Folate-mediated one-carbon pool” (Figure 3D,G and Figure S1C). This striking concordance suggests that Epi4 represents a metabolically hyperactive state where nucleotide biosynthesis is systematically upregulated to sustain rapid cellular replication.

Furthermore, trajectory inference using CytoTRACE identified Epi4 as one of the epithelial states with relatively high inferred developmental potential. Epi4 exhibited a CytoTRACE score comparable to those of Epi2 and Epi5 and higher than those of Epi1 and Epi3, supporting a high-potency malignant phenotype (Figure 3H,I). Consistently, single-cell regulatory network analysis (pySCENIC) and expression profiling demonstrated that Epi4 cells harbor significantly elevated regulon activity of the cell cycle regulator MYBL2, alongside robust expression of canonical proliferation markers such as MKI67 (Figure 3E,F,J–M). Collectively, these multi-dimensional data firmly delineate Epi4 as a hyper-cycling, stem-like reservoir fueled by pyrimidine and folate dependencies within the tumor ecosystem.

#### 2.1.4. Epi4-Derived Folate Efflux Potentially Epigenetically Programs FOLR2^+^ TAMs into an Immunosuppressive Niche via MAFB Activation

Emerging paradigms in tumor ecology demonstrate that intracellular metabolic fluxes do not operate in isolation; instead, they reshape the extracellular metabolome, enabling metabolic intermediates to function as paracrine signals that remodel the immune microenvironment. This prompted us to investigate whether a similar metabolite efflux occurs in tumor cells with a hyperactive pyrimidine state [11,12].

Interrogation of multicellular metabolic crosstalk using MEBOCOST revealed a pronounced communication axis centered on folic acid (vitamin B9) efflux. Global network inference predicted that the Epi4 subpopulation continuously exports folic acid, spatially targeting a macrophage subset that highly expresses the folate receptor FOLR2 (Figure 4A–D).

Notably, FOLR2 expression was not ubiquitous across the myeloid compartment; rather, it was markedly enriched in a tissue-resident-like tumor-associated macrophage (TAM) subpopulation, clearly distinguishing it from pro-angiogenic SPP1^+^ TAMs (Figure 4E–G). Gene set variation analysis (GSVA) consistently demonstrated that FOLR2^+^ and SPP1^+^ TAMs possess fundamentally divergent metabolic and oncogenic landscapes (Figure 4H).

Pathway enrichment analysis revealed that FOLR2^+^ TAMs preferentially channel sequestered folate toward DNA and histone methylation rather than polyamine biosynthesis (Figure 4I,J). Interrogation of rate-limiting enzymes elucidated the mechanistic basis for this metabolic flux divergence: FOLR2^+^ tumor-associated macrophages (TAMs) highly expressed MTR and MAT2A (key drivers of the methionine cycle and intracellular SAM synthesis) alongside several key enzymes involved in histone methylation, whereas ODC1, the rate-limiting enzyme for polyamine synthesis, was virtually absent. Furthermore, the core DNA methyltransferases (DNMT1, DNMT3A, and DNMT3B) were not significantly upregulated (Figure 4K,L). Collectively, these data suggest that Epi4-derived folate is primarily utilized by FOLR2^+^ TAMs to synthesize SAM and predominantly drive histone methylation, thereby fueling global epigenetic reprogramming rather than cellular replication.

To delineate how this folate-driven epigenetic identity translates into functional microenvironmental restructuring, we used CellChat to perform a network analysis of the input and output pathways of FOLR2^+^ TAMs. (Figure S1A). This analysis revealed that FOLR2^+^ TAMs are rewired into dominant signaling hubs, specifically upregulating two classes of pro-tumoral signals: (1) Immunosuppression and tolerance, characterized by the robust predicted secretion of IL-10, TGF-β, GAS6, and galectins, which form a potent network to dampen anti-tumor immunity (Figure S1A). (2) Tissue remodeling and trophic support, acting as a primary source of growth and angiogenic factors (VEGF, EGF, visfatin, GRN, and OSM) to directly fuel tumor proliferation (Figure S1A). Thus, the Epi4–folate axis does not merely provide metabolic sustenance; it is inferred to epigenetically program macrophages into a tolerogenic and highly supportive niche.

To identify the transcriptional executor translating this folate-driven metabolic rewiring into a stable cellular identity, we evaluated transcription factor activities across the myeloid compartment using single-cell regulatory network inference (SCENIC). Unbiased analysis revealed that the regulon of MAFB—a master transcription factor characteristic of tissue-resident macrophages—was profoundly and specifically hyperactivated in FOLR2^+^ TAMs (Figure 4M,N). Given that the folate–methionine cycle dictates the intracellular availability of S-adenosylmethionine (SAM)—the universal methyl donor essential for epigenetic regulation—we posit that this robust folate influx broadly reshapes the epigenetic landscape of these macrophages. This metabolically driven epigenetic remodeling likely establishes a permissive chromatin environment that unlocks and sustains the MAFB transcriptional program. Finally, to assess the functional necessity of this regulatory axis, we performed in silico perturbation using the scTenifoldKnk framework. In silico knockout of either MAFB or FOLR2 predicted disruption of the gene regulatory networks governing canonical tissue-resident markers (e.g., LYVE1 and CD209) (Figure S1B,C).

#### 2.1.5. Spatial Transcriptomics Defines an Epi4–FOLR2^+^ TAM Boundary Niche with a Distance-Dependent Macrophage Phenotype

To characterize the spatial architecture and intercellular dynamics of the Epi4-driven microenvironment in situ, we applied the Xenium high-resolution spatial transcriptomics platform. We first integrated the spatial data with our annotated scRNA-seq reference, achieving robust batch mixing and precise label transfer across the UMAP space (Figure 5A,B).

Mapping these predicted identities back to the physical tissue coordinates faithfully reconstructed the complex spatial distribution of critical subsets (Figure 5C,D). Further examination of Epi4, FOLR2^+^ TAMs and T cells revealed that Epi4 tumor nests and FOLR2^+^ TAMs co-occupied the tumor–stroma margin (Figure 5E).

To dissect the architectural principles of this microenvironment, we performed unsupervised clustering of local neighbourhood composition (Figure 5F,G). Among the identified regions, we focused on Niche 1, a compartment composed predominantly of malignant epithelial cells, and Niche 2, a tumor–myeloid boundary region defined by the concurrent enrichment of Epi4 tumor cells and FOLR2^+^ TAMs (Figure 5G,H). Niche 2 was also closely associated with the vascular endothelium, forming a perivascular arrangement (Figure 5I). Within the peri-tumour compartment, Niche 2 cells were situated closer to the tumor core than T cells (Figure 5J and Figure S1A); this spatial relationship is compatible with reduced T-cell access to the core, but does not establish it. Epi4 cells and FOLR2^+^ TAMs were located at distances compatible with paracrine signaling, consistent with the intercellular crosstalk predicted by MEBOCOST. Because vascular entry sites are a principal route for circulating T cells, we speculate that this perivascular arrangement may contribute to a locally immunosuppressive environment.

Global spatial analysis showed that FOLR2^+^ TAMs were located closer to Epi4 cells than T cells (median distance 65.8 µm versus 97.0 µm; median difference −31.2 µm, 95% confidence interval (CI) −36.8 to −23.8; Figure 5K; null-model statistics in Figure 3). The difference persisted when the analysis was restricted to the proximal microenvironment within a 150 µm radius of Epi4 (median 48.9 µm versus 70.3 µm; −21.4 µm, 95% CI −25.0 to −17.5; Figure 5L; Figure S3C). Within this window, MRC1 and CD163 expression and positivity in FOLR2^+^ TAMs were higher in the far-field (>30 µm) than in the near-field (≤30 µm) region (Figure 5M), and LOESS regression showed that expression of both markers increased with distance from Epi4 across the window (Figure 5N). The two zones differed in cellular composition and not only in folate concentration: the densities of all non-malignant populations were higher beyond 30 µm (T cells 3.8-fold, CAFs 3.6-fold, other myeloid cells 3.4-fold; Figure 5O,P). Collectively, these observations indicate that the M2-associated phenotype of FOLR2^+^ TAMs increases with distance from the Epi4 compartment and that the near-field and far-field zones represent distinct signalling environments; the direction of this gradient is opposite to that predicted by a simple folate-paracrine model, and its determinants remain to be defined.

#### 2.1.6. DTYMK Sustains Tumor Fitness and Drives Folate-Dependent Macrophage Reprogramming

Having characterised the spatial organisation associated with Epi4, we sought to elucidate the intracellular metabolic engine driving this aggressive phenotype. Analysis of metabolic gene expression profiles across epithelial subpopulations revealed that DTYMK—a crucial rate-limiting enzyme and the central intersection hub between both the de novo and salvage pyrimidine biosynthesis pathways—was distinctly hyper-expressed in the aggressive Epi4 subpopulation (Figure 6A,B).

The clinical relevance of this metabolic dependency was robustly validated across extensive patient cohorts: in both the TCGA-BRCA and FUSCC TNBC cohorts, DTYMK expression was profoundly upregulated in tumor tissues compared with normal or adjacent non-tumor counterparts (*p* < 0.001) (Figure 6C,D).

To prospectively evaluate the functional vulnerabilities tied to this metabolic hub, we performed an in silico virtual knockout of DTYMK (Figure 6E–G). Transitioning to in vitro models, we established stable DTYMK knockdown (shDTYMK) in LM2 and EMT6 cell lines (Figure 6H,I). Concordant with our transcriptomic predictions, the depletion of DTYMK profoundly abrogated the oncogenic fitness of these cells, resulting in a dramatic suppression of both overall cell viability and clonogenic formation capacity across multiple targeted sequences (Figure 6J–L).

Having identified DTYMK-associated nucleotide metabolic rewiring as a defining feature of Epi4 cells, we next investigated whether this metabolic state generates extracellular signals capable of remodeling the tumor microenvironment. We first examined whether folate is sufficient to induce macrophage reprogramming. Direct stimulation of macrophages with exogenous folic acid upregulated MAFB and induced an M2 immunoregulatory transcriptional program characterized by robust expression of MRC1, IRF4, and ARG1 (Figure 6M–P).

To determine whether tumor-derived metabolic activity mediates this macrophage phenotype, we performed conditioned-medium experiments. Macrophages cultured with supernatants from control tumor cells effectively sustained this MAFB-associated macrophage transcriptional program. In stark contrast, DTYMK depletion in tumor cells markedly impaired this paracrine capacity, precipitating a dramatic collapse of MAFB, MRC1, IRF4, and ARG1 expression in recipient macrophages. Importantly, supplementation of exogenous folic acid into the DTYMK-deficient conditioned medium completely rescued macrophage reprogramming, demonstrating that folate availability represents the indispensable metabolic mediator downstream of DTYMK-associated tumor rewiring (Figure 6Q–T). Together, these findings establish a potential functional link between the Epi4 metabolic state and macrophage fate determination through a folate-dependent paracrine mechanism.

## 3. Discussion

The role of metabolic crosstalk between cancer cells and immune cells in sculpting an immunosuppressive microenvironment has long been a focal point of oncological research [8,13,14,15]. Here, we identified a DTYMK-associated epithelial metabolic state that potentially remodels the immune microenvironment via folate-dependent signaling to macrophages. These findings underscore the critical importance of evaluating intercellular crosstalk within the tumor microenvironment (TME) when investigating cancer metabolism. Although the present study primarily focuses on the metabolic interplay between tumor cells and macrophages, other cellular constituents of the microenvironment, such as dendritic cells and cancer-associated fibroblasts (CAFs), may play equally pivotal roles in this metabolic network [8].

Folate, a water-soluble B-vitamin, is an indispensable nutrient for numerous critical cellular processes, including one-carbon transfer, cellular methylation, amino acid metabolism, and nucleotide biosynthesis [16]. In the classical paradigm of cancer metabolism, folate-mediated one-carbon metabolism (FOCM) and pyrimidine biosynthesis are predominantly viewed as “intracellular” fuel reservoirs that sustain malignant proliferation and stemness. Consequently, a repertoire of folate metabolism inhibitors, such as methotrexate, has been widely deployed in first-line tumor chemotherapy [17]. However, this conventional cell-autonomous perspective severely underestimates the systemic role of hyperactive metabolic fluxes in sculpting the tumor microenvironment (TME) [17,18]. Emerging studies highlight metabolite efflux as a central driver of ecological remodeling within tumors; for instance, stroma-derived serine fuels tumor growth [19], tumor-secreted lactate provokes T-cell exhaustion [20], and branched-chain ketoacids (BCKAs) released from KRAS-mutant cells directly dictate the immunosuppressive polarization of TAMs [12]. Although the concept of metabolites acting as microenvironmental communication mediators has been validated, it remains a critical scientific blind spot whether folate—the absolute core of one-carbon metabolism—can transcend its purely intracellular metabolic role to function as a specific “paracrine signal” directly orchestrating macrophage remodeling.

Our enrichment analysis revealed that both DNA and histone methylation pathways are concurrently hyperactivated in the FOLR2^+^ TAM subpopulation. This dual enrichment suggests that continuous folate influx saturates the intracellular S-adenosylmethionine (SAM) pool, thereby providing an abundant supply of methyl donors for global epigenetic modifications. However, evaluation of rate-limiting enzymes established that folate-driven macrophage remodeling predominantly relies on histone methylation rather than DNA methylation. Although the enrichment of DNA methylation pathways underscores the sheer abundance of intracellular methyl donors, it is targeted, activating histone modifications that serve as the precise regulatory engine. By establishing a permissive chromatin environment for key transcription factors, histone methylation ultimately dictates and drives the macrophage’s commitment to the M2-polarized core transcriptional network. Indeed, the regulation of macrophage differentiation by histone methylation is well documented. For instance, PRMT1 has been shown to facilitate IL-4-induced M2 macrophage polarization by enhancing H4R3me2a methylation at the PPARγ promoter [21]. Furthermore, ASH1L mediates H3K4me3 methylation to upregulate CCL2 and CSF1 expression, thereby promoting macrophage recruitment and M2 polarization in hepatocellular carcinoma (HCC) [22].

Having delineated the interaction mechanism between Epi4 and FOLR2^+^ TAMs at the single-cell level, we utilized high-resolution Xenium spatial transcriptomics to characterize their topological architecture and functional connectivity in situ. Our results suggest that Epi4 cells and FOLR2^+^ TAMs co-occupy the tumour–stroma margin, with FOLR2^+^ TAMs located closer to Epi4 cells than T cells. This spatial organisation is associated with reduced T-cell density in the adjacent region through microenvironmental remodeling. Classical mathematical models establish that, under ideal conditions, the upper limit for effective intercellular communication via soluble paracrine factors is approximately 250 μm [23]. However, within the solid tumor microenvironment, restricted by dense extracellular matrix, elevated interstitial fluid pressure, and the “sponge capture effect” of high-density receptors, the actual effective diffusion and action radii of small-molecule metabolites and signaling factors are typically compressed to the 100–150 μm range [24,25]. Based on this theoretical framework, we selected 150 μm as the computational window for effective diffusion distance. This allowed for the precise evaluation of relative spatial relationships among distinct cell types, circumventing signal dilution caused by whole-slide cellular inclusion. Furthermore, in accordance with consensus in the spatial multi-omics field—where tumor cells and macrophages typically span 15–20 μm in diameter—we strictly defined 30 μm as the physical threshold for near-field interactions, a range that corresponds to the thickness of one to two adjacent cell layers [26]. Notably, as the observation window shifts to the far-field region (30–150 μm), direct membrane contact is relieved, and cellular phenotypes become predominantly governed by soluble paracrine gradients [27]. Our spatial data revealed a paradoxical distance-dependent phenotypic trajectory: despite being closest to the Epi4 folate source, TAMs in the near-field exhibit suppressed M2 polarization, with the expression of core M2 markers (MRC1 and CD163) exhibiting a marked upward trend as distance from the Epi4 boundary increases. To reconcile this counterintuitive observation, we hypothesize that this near-field zone imposes intense juxtacrine signaling and contact-dependent suppression on TAMs, transiently overriding the M2-polarizing effects of diffused folate. And several non-exclusive explanations also may account for the spatial pattern. First, the near- and far-field zones differ in cellular composition and not only in folate concentration: the near field abuts tumour cells directly, whereas the far field corresponds to the peri-tumour stroma, which contains CAFs and endothelial cells. Stromal and vascular constituents are established sources of macrophage-polarising signals, and a spatially heterogeneous cytokine milieu has been repeatedly linked to macrophage phenotype in tumours [28]. Second, matrix mechanics may contribute. Increased matrix rigidity promotes M2 activation by inducing nuclear deformation and nuclear-pore opening, thereby facilitating nuclear translocation of phosphorylated STAT6 together with increased chromatin accessibility at M2-associated gene promoters, and rigidity-primed macrophages accumulate in stiffened tumours [29]. We did not measure tissue stiffness in this dataset, and we therefore cannot determine whether a rigidity gradient across the 30 µm near-field threshold contributes to the observed pattern. Third, the pattern may not represent a continuum: MRC1 and CD163 are expressed by several macrophage states, and the distance-dependent increase may reflect the spatial segregation of distinct TAM populations—one occupying the tumour interface and another the peri-tumour stroma—rather than a progressive shift along a single polarisation axis. Finally, because this dataset represents a single time point, the direction of the relationship cannot be inferred: the observation is equally compatible with in situ re-programming with distance and with the differential recruitment or retention of pre-existing macrophage states at different positions. This area remains largely unexplored and warrants further investigation. We acknowledge that the M1/M2 classification provides a simplified framework for describing macrophage phenotypes and does not fully capture the substantial heterogeneity and plasticity of tumor-associated macrophages (TAMs). Although we used established M1/M2 marker sets to facilitate comparison with previous studies, these markers should be interpreted as descriptive rather than definitive assignments of macrophage states. Future studies using higher-resolution transcriptional and functional profiling will be needed to further characterize the macrophage states induced by tumour-derived signals.

Depletion of the metabolic hub DTYMK impairs overall tumor cell proliferation, which in turn could introduce non-specific secondary changes to the global secretome. Our functional assays instead point to a specific dependence on folate. Because we normalized tumor conditioned medium strictly to live-cell counts, the confounding effect of altered proliferation rates was minimized. Adding a single exogenous metabolite, folic acid, was sufficient to fully rescue the M2-associated macrophage phenotype in DTYMK-deficient contexts. This indicates that folate is the paracrine mediator downstream of this metabolic rewiring, and that the phenotype is not simply a consequence of generalized tumor cell deterioration.

Several limitations should be considered. First, our inference of metabolite-mediated communication rests on gene expression: MEBOCOST estimates metabolite levels from the expression of enzymes annotated as producing or consuming them and derives sender–receiver relationships from inferred efflux/influx and sensor expression, without measuring metabolites, secretion or flux. The resulting scores are relative, transcriptome-derived rankings; their significance reflects non-randomness given the expression data rather than demonstrated transport, and the method was benchmarked at presence/absence rather than quantitative accuracy. Neither folate nor S-adenosylmethionine (SAM) was quantified, and the route by which folate would leave Epi4 cells was not identified: folate is largely retained intracellularly in polyglutamated form, and no candidate exporter was tested. Second, the downstream steps of the axis are inferred rather than measured: FOLR2-dependent uptake, methyl-donor availability and histone methylation were not examined directly, and MAFB was identified computationally (pySCENIC) and by expression changes in vitro, so its regulatory role in establishing the FOLR2^+^ TAM phenotype remains to be defined. Whether FOLR2^+^ TAMs are immunosuppressive in this tumour context, or instead support T-cell responses as reported elsewhere, will require functional testing. Third, our experimental support is confined to cell culture: although folate was sufficient to induce the macrophage phenotype and largely restored it after DTYMK depletion, the rescue used a supraphysiological folate concentration, no selective DTYMK inhibitor was tested, and no in vivo experiment was performed. Fourth, the spatial analysis rests on a single section, and myeloid identities there were assigned by label transfer from the matched scRNA-seq reference because FOLR2 is absent from the 313-gene panel; the spatial organization we describe cannot be recapitulated in culture. In the absence of *Folr2* assessment in iBMMs and of FOLR2 blockade, the axis is best regarded as a human-derived hypothesis with partial functional support that must be confirmed in human macrophages before clinical translation. Future studies using patient-derived organoid co-culture or syngeneic mouse models with conditional Dtymk knockout, together with spatially resolved metabolomics, SAM and histone-mark quantification, and targeted FOLR2 and MAFB perturbation, will be needed to determine whether DTYMK targeting alters the organization of the tumor–myeloid boundary region.

## 4. Materials and Methods

### 4.1. Data Acquisition and Processing

Bulk RNA sequencing profiles and corresponding clinical information for Breast Cancer (BRCA) were obtained from The Cancer Genome Atlas (TCGA) using the “TCGAbiolinks” (v2.34.1) R package [30]. Additionally, bulk transcriptomic profiles for the FUSCC-TNBC cohort were acquired from the GEO dataset GSE118527 [31]. Single-cell RNA sequencing (scRNA-seq) data derived from human BRCA tissues were acquired from the Gene Expression Omnibus (GEO) dataset GSE176078 [32]. The “Seurat” R package (v5.3.1) was employed for downstream scRNA-seq data processing [33,34,35,36,37]. Data were normalized using the NormalizeData function, and highly variable features were identified utilizing FindVariableFeatures (nfeatures = 3000). Dimensionality reduction was performed via Principal Component Analysis (PCA) based on 10 principal components. Uniform Manifold Approximation and Projection (UMAP) and t-distributed Stochastic Neighbor Embedding (t-SNE) [38] algorithms were utilized for cell dimensionality reduction and visualization. Cell identities were annotated based on canonical marker expression, and cluster-specific marker genes were identified using the FindAllMarkers function. For epithelial sub-clustering, the cancer epithelial compartment was isolated and subjected to an unsupervised re-clustering pipeline following identical normalization and dimensionality reduction procedures.

### 4.2. Functional Enrichment and Metabolic Landscape Profiling

Gene Set Variation Analysis (GSVA) was performed utilizing the “GSVA” R package (v2.0.5) [39] to evaluate the enrichment of MSigDB Hallmark gene sets [40] and specific biological pathways in both scRNA-seq subpopulations and TCGA-BRCA bulk cohorts. Functional enrichment analysis based on the Gene Ontology (GO) [41] was performed utilizing the “clusterProfiler” (v4.14.4) [42,43], and GO terms with a Benjamini–Hochberg adjusted *p*-value < 0.05 were considered significantly enriched; the top enriched terms were visualized with dot plots and bar plots using the “enrichplot” R package(v 1.26.6). The metabolic landscape at single-cell resolution was quantified using the “scMetabolism” R package (v0.2.1) [44,45], enabling the mapping of pathway activity distribution profiles. In addition, Gene Set Enrichment Analysis (GSEA) [46] was performed on the differentially expressed genes (DEGs) ranked by log2 fold change against the MSigDB Hallmark gene set collection [40], and gene sets with a false discovery rate (FDR) q-value < 0.25 were considered significantly enriched, with the normalized enrichment score (NES) reported. M1 and M2 gene-set enrichment scores were calculated using AddModuleScore implemented in Seurat. The “classically activated” (M1) signature comprised CCL5, CCR7, CD40, CD86, CXCL9, CXCL10, CXCL11, IDO1, IL1A, IL1B, IL6, IRF1, IRF5 and KYNU (14 genes); the “alternatively activated” (M2) signature comprised CCL4, CCL13, CCL18, CCL20, CCL22, CD276, CLEC7A, CTSA, CTSB, CTSC, CTSD, FN1, IL4R, IRF4, LYVE1, MMP9, MMP14, MMP19, MSR1, TGFB1, TGFB2, TGFB3, TNFSF8, TNFSF12, VEGFA, VEGFB and VEGFC (27 genes) [47]. Scores are relative quantities that depend on the gene set used and are not absolute measures of macrophage activation state.

### 4.3. Cell–Cell Communication and Transcription Factor Analysis

Intercellular communication networks were inferred and visualized using the “CellChat” R package (v1.6.1) [48] based on the “CellChatDB.human” database. Furthermore, metabolite-sensor crosstalk was predicted using MEBOCOST (v1.2.2) [49]. Interaction scores were calculated by multiplying the inferred metabolite efflux potential of sender cells by the sensor expression of receiver cells. Significance was determined via a permutation test (1000 randomizations, *p* < 0.05). Transcription-factor regulatory network analysis was carried out via the pySCENIC package(v0.12.1) [50].

### 4.4. Single-Cell Trajectory Inference and Virtual Knockout Modeling

The developmental potential and hierarchical state of epithelial subpopulations were predicted using the “CytoTRACE” R package (v0.3.3) [51]. To validate the functional requirement of key genes, in silico perturbation modeling was executed utilizing the “scTenifoldKnk” R package (v1.0.1) [52]. Virtual gene knockouts were simulated by constructing single-cell gene regulatory networks (scGRNs) via quasi-manifold alignment, enabling the prediction of systemic transcriptional collapse and downstream perturbed targets following the simulated deletion of specific genes.

### 4.5. Spatial Transcriptomics Analysis

The Xenium spatial transcriptomics dataset for human breast cancer (Xenium_FFPE_Human_Breast_Cancer_Rep1_outs) was publicly acquired from 10x Genomics (https://www.10xgenomics.com/datasets, accessed on 29 April 2026). This dataset originates from an ER^+^/PR^−^/HER2^+^ stage II-B invasive ductal carcinoma, with two FFPE blocks; we analyzed one tissue section. Raw data were subjected to quality control and normalization utilizing the OmicVerse [53] and scanpy [54] frameworks. Cells with fewer than 10 detected transcripts were filtered out. By employing the ingest algorithm, fine-grained annotations from a matched scRNA-seq reference dataset were mapped onto physical coordinates, enabling the precise identification of high-resolution cellular subpopulations. To decode the spatial architecture, squidpy [55] was used to construct a cellular graph network and compute local neighborhood composition features, facilitating the definition of spatial niches with specific biological functions via unsupervised clustering. Finally, distances from every cell to the nearest tumor-core (Niche 1) cell and to the nearest Epi4 cell were computed using a k-d tree (scipy.spatial.cKDTree, k = 1); these are centroid-to-centroid distances and do not represent cell-boundary distances. Effect sizes, 95% confidence intervals, and significance for the differences between cell populations were obtained with a spatially blocked permutation null model that accounts for the spatial clustering of cells.

### 4.6. Cell Culture and Lentiviral Transduction

Human embryonic kidney 293T (HEK293T) cells, immortalized bone marrow-derived macrophages (iBMMs), human LM2 and the murine breast cancer cell line EMT6 were obtained from the American Type Culture Collection (ATCC; Manassas, VA, USA). Cell line identities were authenticated via short tandem repeat (STR) profiling, and routine screening was performed to ensure the absence of mycoplasma contamination. All cell lines were cultured in Dulbecco’s Modified Eagle Medium (DMEM; Gibco, New York, NY, USA, cat# C11995500BT) supplemented with 10% fetal bovine serum (FBS) and 1% penicillin/streptomycin at 37 °C in a humidified 5% CO_2_ incubator.

### 4.7. Plasmid Construction and Lentivirus Infection

Target sequences for DTYMK short hairpin RNA (shRNA) were designed based on the Sigma-Aldrich shRNA database. These core shRNA fragments were subsequently inserted into the pLKO.1-mCherry lentiviral backbone. For lentiviral packaging, HEK293T cells were co-transfected with the engineered core vectors and the packaging plasmids psPAX2 (Addgene, Cambridge, MA, USA, cat#12260) and pMD2.G (Addgene, cat#12259) at a mass ratio of 5:3:2. Forty-eight hours post-transfection, viral supernatants were harvested and clarified through a 0.45-μm filter. Target tumor cells were transduced with the viral particles for 12 h in the presence of 10 μg/mL polybrene (Beyotime, Shanghai, China, cat#C0351). Transduced cells stably expressing mCherry were purified via three consecutive rounds of fluorescence-activated cell sorting (FACS) utilizing a MoFlo Astrios system (Beckman Coulter, Brea, CA, USA). Primer sequences used for plasmid construction are detailed in Table S1.

### 4.8. Western Blotting Analysis

Whole-cell lysates were prepared using RIPA lysis buffer (Sangon Biotech, Shanghai, China, cat#C500005-0100) supplemented with the Halt™ Protease and Phosphatase Inhibitor Cocktail (ThermoFisher, Waltham, MA, USA). Protein yields were quantified using a BCA protein assay kit (Solarbio, Beijing, China, cat#PC0020). Samples were subsequently mixed with 5× loading buffer (NCM Biotech, Suzhou, China) and denatured by boiling at 100 °C for 10 min. Equal quantities of total protein were resolved via 10% SDS-PAGE and electroblotted onto PVDF membranes using a Trans-Blot Turbo transfer system (Bio-Rad, Hercules, CA, USA). Nonspecific binding sites were blocked by incubating the membranes in phosphate-buffered saline (PBS) containing 0.1% Tween-20 (PBST) and 5% non-fat dry milk. The membranes were then probed overnight at 4 °C with specific primary antibodies against DTYMK (ABclonal, Wuhan, Hubei, China, cat#A6370, 1:1000) and GAPDH (Signalway Antibody, Frederick, MD, USA, cat#21612, 1:10,000). Following extensive washing in PBST, the blots were incubated with HRP-conjugated anti-rabbit secondary antibodies (Abcam, Cambridge, UK) for 1 h at room temperature. Immunoreactive bands were visualized using the Pierce ECL Western Blotting Substrate (ThermoFisher, Waltham, MA, USA) and captured on a ChemiDoc™ XRS+ Imaging System (Bio-Rad).

### 4.9. Cell Viability and Colony Formation Assays

Cell proliferation was assessed using the Cell Counting Kit-8 (CCK-8) assay (CCK-8, cat#C0037, Beyotime Biotechnology, Shanghai, China). Briefly, cells were seeded into 96-well plates, and CCK-8 reagent was added at designated time points. The absorbance was measured at 450 nm using a microplate reader. For colony formation assays, cells were seeded in 6-well plates with 2000 cells per well and cultured for 1–2 weeks. Colonies were then fixed with 4% paraformaldehyde, stained with crystal violet (Sangon Biotech, Shanghai, China, cat#A600331), and macroscopic colonies were counted to evaluate clonogenic survival.

### 4.10. Preparation of Normalized Tumor Conditioned Medium (TCM)

Control and DTYMK-knockdown EMT6 cells at 70% confluence were washed three times with PBS to eliminate basal folate and cultured in folate-free RPMI 1640 (Gibco™, Catalog No. 27016021) supplemented with dialyzed FBS (Cellco, Catalog No. FBS007). After 48 h of culture, the TCM was collected, sequentially centrifuged (300× *g* for 5 min; 2000× *g* for 10 min at 4 °C) to remove cellular debris, and sterile-filtered (0.22 µm PES). To ensure metabolic normalization across groups, the remaining adherent cells were quantified. The TCM from the higher-yield group was then proportionally diluted with fresh folate-free medium to match the live-cell count of another group.

### 4.11. Macrophage Co-Culture and Folate Rescue

Normalized TCM from control and shDTYMK EMT6 cells was prepared as described above. iBMMs were seeded and cultured until adherence, then washed three times with PBS to remove residual medium and basal folate. For co-culture experiments, iBMMs were cultured for 48 h in medium containing 50% normalized TCM; in parallel, for the folate rescue experiments, iBMMs were cultured in folate-free RPMI 1640 supplemented with dialyzed FBS in the absence of TCM. In both conditions, the medium was supplemented with either 0.02% DMSO (vehicle control) or folic acid at a final concentration of 10 μM (cat# S4605, Selleck Chemicals LLC, Houston, TX, USA). After 48 h of stimulation, all cells were harvested, total RNA was extracted using TRIzol reagent (Invitrogen, Carlsbad, MA, USA, cat# 15596018CN), and the expression of canonical M2 macrophage markers was quantified via RT-qPCR, with TBP as the internal reference.

### 4.12. RT-qPCR

Total RNA was isolated from cultured cells using TRIzol reagent (Invitrogen, Carlsbad, MA, USA, cat# 15596018CN), and RNA concentration and purity were assessed via a NanoDrop spectrophotometer. Complementary DNA (cDNA) was synthesized employing the PrimeScript RT reagent Kit with gDNA Eraser (Takara, Kusatsu, Japan, cat#RR047A). Quantitative real-time PCR (RT-qPCR) was executed on a Real-Time PCR System (ThermoFisher, Waltham, MA, USA) utilizing the TB Green Advantage qPCR Premix (Takara, cat#639676). Gene-specific primer sequences are provided in Table S2. Relative mRNA abundance was normalized to the housekeeping gene TBP, and fold changes were calculated according to the 2^−ΔΔCt^ method.

### 4.13. Statistical Analysis

All statistical analyses were conducted in the R software environment (v4.4.1). Differences in continuous variables between two independent groups were assessed using the Wilcoxon rank-sum test or Student’s *t*-test, as appropriate. To quantify the magnitude of metabolic pathway activity differences between malignant and normal epithelial cells, Cohen’s d effect sizes and their 95% confidence intervals (CIs) were calculated using a custom R function, with CIs derived from the noncentral t-distribution [56]. A *p*-value of <0.05 was considered to indicate statistical significance.

## 5. Conclusions

In conclusion, our study describes a metabolically driven epigenetic crosstalk network in TNBC and provides within-section spatial evidence that the Epi4/FOLR2^+^ TAM organization is associated with reduced T-cell density. We reveal that the pyrimidine-hyperactive state of tumor epithelial cells, sustained by the rate-limiting enzyme DTYMK, extends far beyond tumor-intrinsic fitness to function as a potent intercellular communication axis. By continuously exporting folate, these malignant cells are proposed to epigenetically reprogram local FOLR2^+^ macrophages via a SAM–histone methylation–MAFB cascade, locking them into a highly immunosuppressive and tissue-remodeling fate. Critically, this metabolic symbiosis manifests spatially as a perivascular region with reduced T-cell density, consistent with an immune-exclusion phenotype. By identifying DTYMK as a metabolic dependency that sustains this niche in our model systems, our findings nominate a candidate vulnerability. Whether it can be exploited therapeutically, and whether it would restore immune access or improve immunotherapy responses, remains to be determined in vivo.

## Figures and Tables

**Figure 1:**
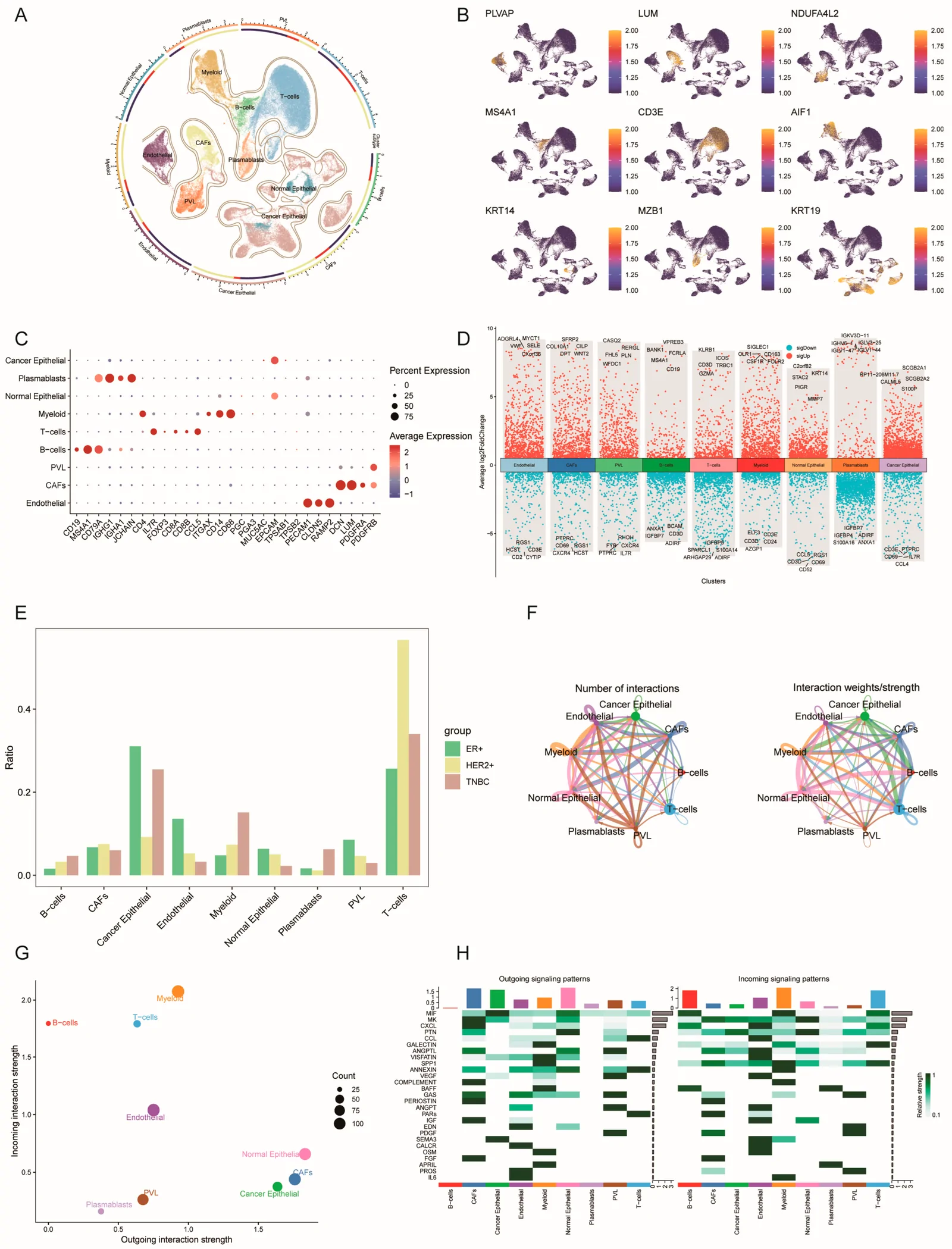
Single-Cell Landscape Reveals a Dominant Epithelial-to-Myeloid Communication Axis in the Tumor Microenvironment. (**A**) Global Uniform Manifold Approximation and Projection (UMAP) integrated with a circular stacked bar chart illustrating the transcriptomic distribution and subtype-specific proportions of major cellular compartments. (**B**,**C**) Feature plots and a comprehensive dot plot validating the precise expression of canonical marker genes defining each distinct cell identity. (**D**) Scatter plot highlighting the top differentially expressed marker genes driving the transcriptional distinctiveness of each cellular cluster. (**E**) Grouped bar plot quantifying the relative compositional abundance of each identified cell type across ER^+^, HER2^+^, and TNBC molecular subtypes. (**F**) CellChat-inferred interactome networks visualizing the total number of interactions and global interaction weights among diverse cell populations. (**G**) 2D scatter plot evaluating the dominant roles of specific cell types within the microenvironment based on their aggregate outgoing and incoming communication strengths. (**H**) Heatmaps characterizing the detailed outgoing and incoming signaling pathway patterns orchestrating the intercellular crosstalk.

**Figure 2:**
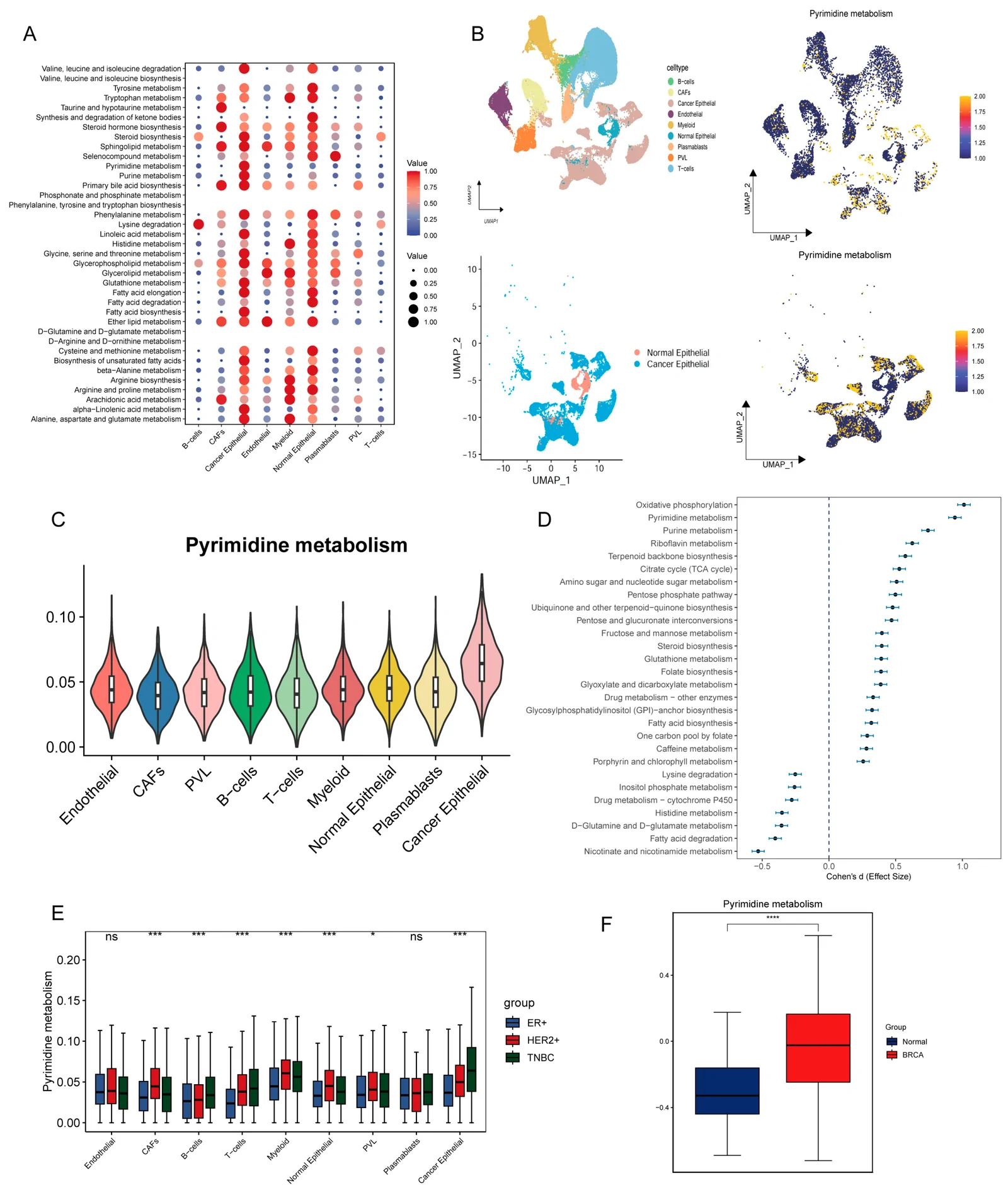
Metabolic Profiling Uncovers Pyrimidine Hypermetabolism as a Defining Epithelial Hallmark in TNBC. (**A**) Dot plot profiling the comprehensive metabolic pathway activities across distinct cellular compartments within the tumor microenvironment. (**B**) UMAPs revealing the specific spatial enrichment of pyrimidine metabolism within the cancer epithelial population. (**C**) Violin plot quantifying single-cell metabolic scores, confirming the peak activation of pyrimidine metabolism in cancer epithelial cells. (**D**) Forest plot of Cohen’s d effect sizes highlighting pyrimidine metabolism as a top upregulated pathway in tumor versus normal epithelia. (**E**) Grouped box plot of pyrimidine pathway scores across the nine major cellular compartments, stratified by ER^+^, HER2^+^, and TNBC molecular subtype. Statistical significance was assessed using the Kruskal–Wallis test. (**F**) Box plot showing pyrimidine pathway scores in BRCA tumor tissues compared with normal samples. * *p* < 0.05, *** *p* < 0.001, **** *p* < 0.0001; ns, not significant.

**Figure 3:**
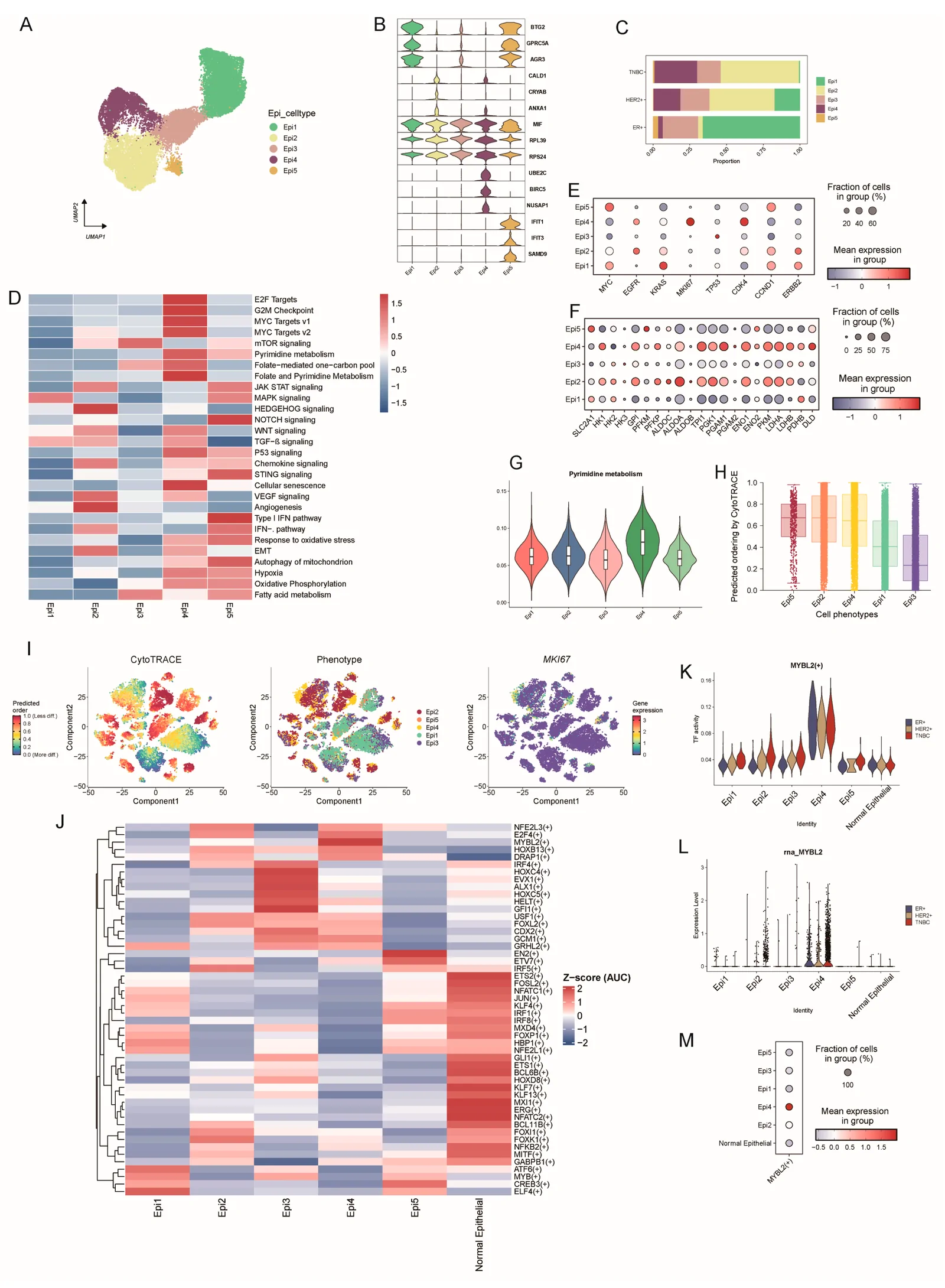
Identification of Epi4, a High-Potency, Hyper-Proliferative Subpopulation Driven by Coupled Pyrimidine and Folate Dependencies. (**A**,**B**) UMAP and corresponding expression violin plots delineating five distinct tumor epithelial subpopulations (Epi1–Epi5). (**C**) Stacked bar plot illustrating the proportional distribution of these epithelial subclusters across distinct BRCA molecular subtypes. (**D**) GSVA heatmap highlighting the differential activation of Hallmark pathways, emphasizing the proliferative and metabolic rewiring in Epi4. (**E**,**F**) Dot plots evaluating the specific expression patterns of key genes associated with tumor stemness and glycolysis. (**G**) Violin plot quantifying the specifically elevated pyrimidine metabolism activity within the Epi4 subpopulation. (**H**,**I**) CytoTRACE developmental potential scoring (bar plot) and t-SNE projection confirming the high-potency state of Epi4. (**J**–**M**) pySCENIC regulon activity heatmap and corresponding violin plots identifying MYBL2 as a dominant transcription factor driving the Epi4 phenotype.

**Figure 4:**
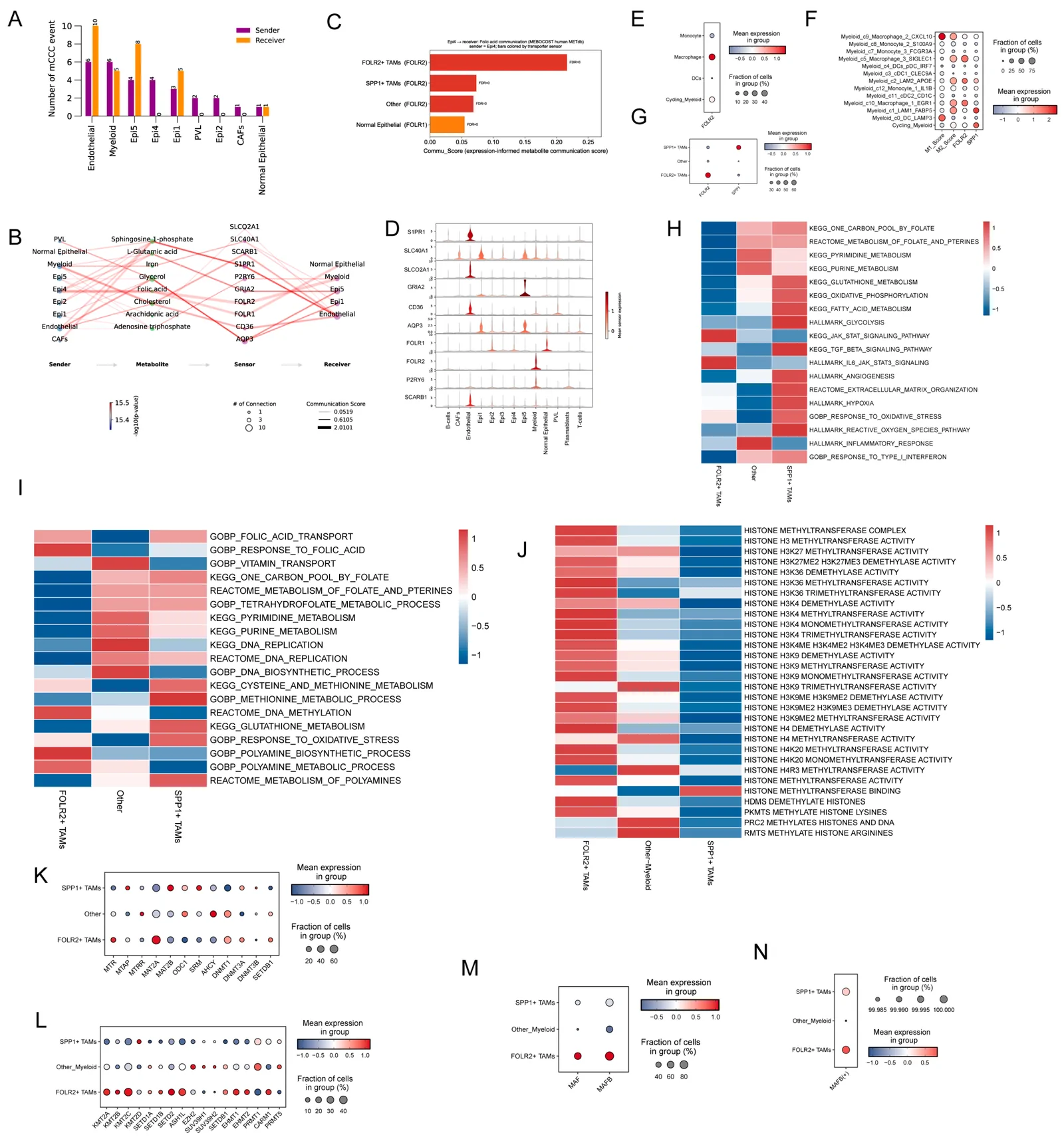
Epi4-Derived Folate Efflux Epigenetically Programs FOLR2^+^ TAMs into an Immunosuppressive Niche via MAFB Activation. (**A**) Bar plot summarizing the total number of inferred metabolic cell–cell communication (mCCC) events for each cell type acting as senders or receivers. (**B**) Sankey diagram detailing the mCCC network, emphasizing the dominant Epi4-derived folic acid axis targeting myeloid FOLR2 receptors. (**C**) Horizontal bar plot quantifying the MEBOCOST communication scores of the Epi4-derived folic acid efflux axis. (**D**) Violin plots quantifying the expression levels of distinct metabolite receptors among individual cell types. (**E**–**G**) Dot plots validating the expression levels of FOLR2 and SPP1, alongside M1 M2 polarization scores in distinct myeloid cell subpopulations. (**H**) Heatmap of GSVA scores revealing divergent metabolic and oncogenic pathway activities between FOLR2^+^ and SPP1^+^ TAMs. (**I**,**J**) Targeted GSVA heatmap demonstrating that FOLR2^+^ TAMs preferentially channel internalized folate towards histone methylation. (**K**,**L**) Dot plots visualize the expression levels of pivotal enzymes from folate-associated pathways and histone methyltransferases within the three subpopulations. (**M**,**N**) Dot plot showing the specific upregulation of the lineage-determining transcription factor MAFB and its regulon in FOLR2^+^ TAMs.

**Figure 5:**
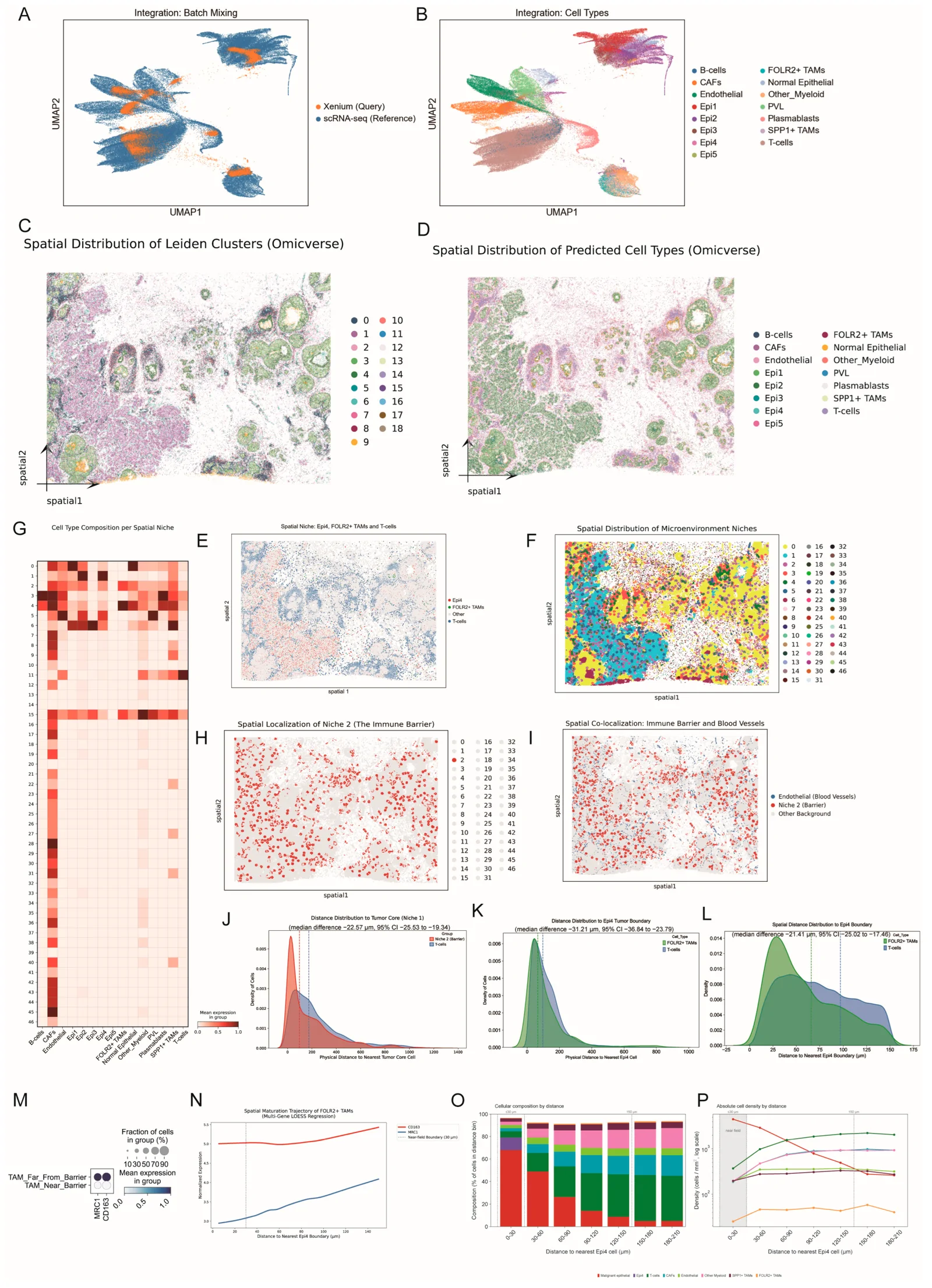
Spatial Transcriptomics Defines an Epi4–FOLR2^+^ TAM Boundary Niche with a Distance-Dependent Macrophage Phenotype. (**A**) UMAP plot demonstrating robust batch mixing between the Xenium spatial query and the scRNA-seq reference data. (**B**) UMAP showing the successful annotation transfer of cell types to the spatial dataset. (**C**) Spatial map displaying the physical distribution of unsupervised Leiden clusters across the tissue. (**D**) In situ physical mapping of the predicted high-resolution cell types. (**E**) Spatial isolation highlighting the specific distribution of Epi4 cells, FOLR2^+^ TAMs, and T-cells. (**F**) Spatial mapping delineating distinct microenvironmental niches across the tissue architecture. (**G**) Heatmap quantifying the cellular composition proportions within each identified spatial niche. (**H**) Physical mapping of Niche 2, representing the spatial localization of Niche 2. (**I**) Spatial co-localization analysis demonstrating that the Niche 2 barrier is closely associated with endothelial blood vessels. (**J**) Density plot revealing that FOLR2^+^ TAMs localize closer to the tumor core than T-cells. Median distance to the nearest Niche 1 cell: 39.51 µm for Niche 2 cells versus 62.08 µm for T cells (median difference −22.57 µm, 95% CI −25.53 to −19.34, block bootstrap). (**K**) Density plot highlighting the tight enrichment of FOLR2^+^ TAMs at the Epi4 boundary compared with T cells. Median distance to the nearest Epi4 cell: 65.80 µm for FOLR2^+^ TAMs versus 97.01 µm for T cells (−31.21 µm, 95% CI −36.84 to −23.79). (**L**) Same comparison restricted to cells within 150 µm of the Epi4 boundary: 48.89 µm versus 70.31 µm (−21.41 µm, 95% CI −25.02 to −17.46). Confidence intervals were obtained by spatial block bootstrap and account for the spatial clustering of cells; permutation null-model statistics and design effects are given in Figure S2. (**M**) Dot plot showing significantly upregulated M2 markers (CD163, MRC1) in TAMs localized far from the barrier. (**N**) Locally estimated scatterplot smoothing (LOESS) regression curve demonstrating the spatial maturation trajectory of FOLR2^+^ TAMs dependent on their proximity to the Epi4 boundary. (**O**) Cell-type composition as a function of distance from the nearest Epi4 cell. (**P**) Cell density as a function of distance from the nearest Epi4 cell.

**Figure 6:**
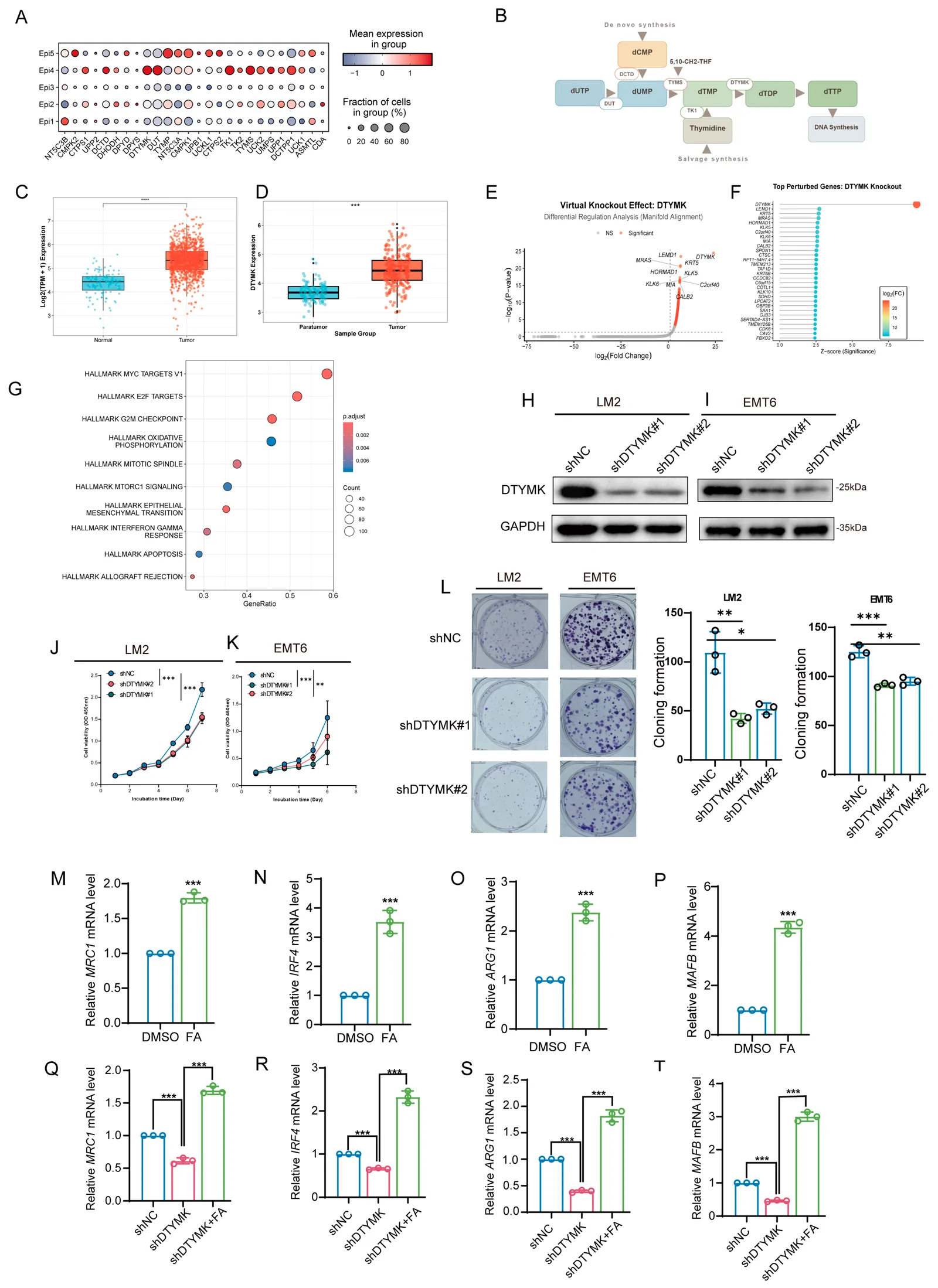
DTYMK sustains tumor fitness and drives folate-dependent macrophage reprogramming. (**A**,**B**) Metabolic gene expression profiling across epithelial subpopulations, highlighting the distinct upregulation of DTYMK—a rate-limiting enzyme bridging de novo and salvage pyrimidine biosynthesis—in the aggressive Epi4 subpopulation. (**C**,**D**) Validation of DTYMK clinical relevance, showing significant upregulation in tumor tissues compared with normal or adjacent non-tumor counterparts in the TCGA-BRCA (**C**) and Fudan University Shanghai Cancer Center (FUSCC) triple-negative breast cancer (TNBC) cohort (**D**) cohorts. (**E**–**G**) In silico virtual knockout of DTYMK. Differential regulation analysis (**E**), top perturbed genes (**F**), and Gene Set Enrichment Analysis (GSEA) (**G**) predict that DTYMK perturbation collapses networks essential for tumor stemness and proliferation. (**H**,**I**) Immunoblot analysis confirming stable DTYMK knockdown (shDTYMK#1 and shDTYMK#2) relative to the negative control (shNC) in LM2 (**H**) and EMT6 (**I**) cell lines. (**J**–**L**) Cell viability assays demonstrating that DTYMK knockdown markedly suppresses the overall proliferation of LM2 (**J**) and EMT6 (**K**) cells, accompanied by a significant reduction in clonogenic formation capacity (**L**). (**M**–**P**) Macrophage reprogramming induced by direct stimulation with exogenous folic acid (FA) versus dimethyl sulfoxide (DMSO) vehicle control. Exogenous FA alone is sufficient to upregulate MAFB (**P**) and the M2 immunoregulatory markers MRC1 (**M**), IRF4 (**N**), and ARG1 (**O**). (**Q**–**T**) Conditioned-medium co-culture assays assessing the paracrine role of tumor metabolic activity. DTYMK depletion in tumor cells markedly impairs their paracrine capacity, causing a substantial suppression of MRC1 (**Q**), IRF4 (**R**), ARG1 (**S**), and MAFB (**T**) expression in recipient macrophages. Data are presented as mean ± SD. Statistical significance was assessed using an unpaired two-tailed Student’s *t* test. *n* = 3 biologically independent replicates per group. * *p* < 0.05, ** *p* < 0.01, *** *p* < 0.001, **** *p* < 0.0001, ns, not significant.

## Data Availability

All datasets analyzed during the current study are publicly available. Bulk and single-cell transcriptomic datasets can be accessed in the NCBI Gene Expression Omnibus (GEO) database under accession numbers GSE118527 and GSE176078, respectively. The breast cancer (BRCA) bulk RNA sequencing profiles and corresponding clinical data are available through The Cancer Genome Atlas (TCGA) program via the Genomic Data Commons (GDC) Data Portal. The Xenium spatial transcriptomics dataset is accessible in the 10x Genomics public repository (https://www.10xgenomics.com/datasets, accessed on 29 April 2026). Further details regarding data acquisition and processing are provided in the Materials and Methods section.

## Supplementary Materials

The following supporting information can be downloaded at: https://www.mdpi.com/article/10.3390/ijms27198626/s1.

## Abbreviations

The following abbreviations are used in this manuscript:

ATCC	American Type Culture Collection
BCA	Bicinchoninic acid
BRCA	Breast cancer
BCKAs	Branched-chain ketoacids
CAF	Cancer-associated fibroblast
CCK-8	Cell Counting Kit-8
cDNA	Complementary DNA
CI	Confidence interval
DEG	Differentially expressed gene
DMEM	Dulbecco’s Modified Eagle Medium
DMSO	Dimethyl sulfoxide
DTYMK	Deoxythymidylate kinase
ECL	Enhanced chemiluminescence
FACS	Fluorescence-activated cell sorting
FBS	Fetal bovine serum
FDR	False discovery rate
FOLR2	Folate receptor 2
FUSCC	Fudan University Shanghai Cancer Center
FOCM	Folate-mediated one-carbon metabolism
GDC	Genomic Data Commons
GEO	Gene Expression Omnibus
GO	Gene Ontology
GSEA	Gene set enrichment analysis
GSVA	Gene set variation analysis
HRP	Horseradish peroxidase
iBMM	Immortalized bone marrow-derived macrophage
LOESS	Locally estimated scatterplot smoothing
mCCC	Metabolic cell–cell communication
NES	Normalized enrichment score
PCA	Principal component analysis
PBS	Phosphate-buffered saline
PBST	Phosphate-buffered saline with Tween-20
PDO	Patient-derived organoid
PVDF	Polyvinylidene fluoride
qPCR	Quantitative polymerase chain reaction
RNA	Ribonucleic acid
RPMI	Roswell Park Memorial Institute
RT-qPCR	Reverse transcription quantitative polymerase chain reaction
SAM	S-Adenosylmethionine
scGRN	Single-cell gene regulatory network
scRNA-seq	single-cell RNA sequencing
SCENIC	Single-cell regulatory network inference and clustering
SDS-PAGE	Sodium dodecyl sulfate–polyacrylamide gel electrophoresis
shRNA	Short hairpin RNA
STR	Short tandem repeat
TAM	Tumor-associated macrophage
TCGA	The Cancer Genome Atlas
TCM	Tumor-Conditioned Medium
TME	Tumor microenvironment
TNBC	Triple-negative breast cancer
UMAP	Uniform manifold approximation and projection
